# Endocrine responses and food intake in fasted individuals under the influence of glucose ingestion

**DOI:** 10.1371/journal.pone.0211514

**Published:** 2019-01-25

**Authors:** Janis Marc Nolde, Jana Laupenmühlen, Arkan Al-Zubaidi, Marcus Heldmann, Thomas F. Münte, Kamila Jauch-Chara

**Affiliations:** 1 Department of Neurology, University of Lübeck, Lübeck, Germany; 2 Department of Psychiatry, University of Lübeck, Lübeck, Germany; 3 Department of Psychiatry, University of Kiel, Kiel, Germany; University of Pisa, ITALY

## Abstract

**Introduction:**

Different metabolic conditions can affect what and how much we eat. Hormones of glucose metabolism and adipokines such as adiponectin take part in the control of these decisions and energy balance of the body. However, a comprehensive understanding of how these endocrine and metabolic factors influence food intake has not been reached. We hypothesised that the amount of food a person consumes differs substantially after a fasting period even after the energy deficit was partially removed by glucose ingestion and endocrine signals like insulin and C-peptide indicated a high glucose metabolic status. Furthermore, the macronutrient composition of the consumed food and a possible association with adiponectin under the influence of glucose ingestion was assessed.

**Methods:**

In a within-subject design, 24 healthy males participated in both a fasting (42 h) and control (non-fasting) condition. A total of 20 blood samples from each subject were collected during each condition to assess serum levels of adiponectin, insulin, C-peptide, cortisol and ACTH. At the end of each condition food intake was measured with an ad libitum buffet after the acute energy deficit was compensated using a carbohydrate-rich drink.

**Results:**

The total amount of caloric intake and single macronutrients was higher after the fasting intervention after replenishment with glucose. All recorded hormone levels, except for adiponectin, were significantly different for at least one of the study intervals. The relative proportions of the macronutrient composition of the consumed food were stable in both conditions under the influence of glucose ingestion. In the non-fasting condition, the relative amount of protein intake correlated with adiponectin levels during the experiment.

**Discussion and conclusion:**

An anabolic glucose metabolism after glucose ingestion following a fasting intervention did not even out energy ingestion compared to a control group with regular food intake and glucose ingestion. Anorexigenic hormones like insulin in this context were not able despite higher levels than in the control condition to ameliorate the drive for food intake to normal or near normal levels. Relative macronutrient intake remains stable under these varying metabolic conditions and glucose influence. Serum adiponectin levels showed a positive association with the relative protein intake in the non-fasting condition under the influence of glucose although adiponectin levels overall did not differ in between the conditions.

## Introduction

Both obesity and its associated metabolic disorders have become a global health burden in recent years [1]. Excessive calorie intake and macronutrient composition of consumed food are major factors in the development of this obesity pandemic [2–5]. Therefore, investigation into the quantity and food composition an individual consumes and why may assist in developing a more comprehensive understanding of how metabolic disorders develop.

The amount of food we consume is regulated by a balance of orexigenic and anorexigenic influences on areas of the central nervous system [6]. This balance is determined by a large number of interneuronal and endocrine signalling substances [7–9]. How these factors are computed in the resulting hunger, satiety and overall energy intake and which factors are the most dominant in this equation is not well understood. Hormones involved in the control of glucose metabolism are likely to be part of these crosslinks, and insulin is understood to be one of the factors that act as an anorexigenic signal [10].How big the influence of single aspects of these regulatory processes like hormones of glucose metabolism is remains uncertain.

Previous studies in rodents found differences in food selection after short fasting periods [11], however it has not yet been investigated whether the same would occur in humans after the acute energy shortage has been partly compensated. Short-term energy status of the body is reflected by hormones like insulin and C-peptide while corticosteroids play a fundamental role in control of glucose metabolism. To which degree the physiological release of insulin as a response to raising glucose levels in the blood after ingestion of carbohydrates can ameliorate feelings of hunger is unknown.

While the quantity of food consumed appears to be the central factor in development of obesity [5], the quality of its components seems to wield some influence as well. Studies suggest that the composition of macronutrients an individual chooses before initiating a therapeutic diet has implications for the diet’s success [12] and similarly that the composition of the therapeutic diet influences diet outcomes and physiological parameters [13,14]. Furthermore, it has been shown that the variation in macronutrient composition of consumed food results in different hormonal responses [15] which might regulate satiety and hunger signals, as well as the choice of macronutrients one prefers. One of these hormones is adiponectin whose serum levels vary after diets as a function of fat and carbohydrate content of the diet [16,17]. Other hormones associated with glucose metabolism, such as insulin [18] and cortisol [19,20], have also been found to be influenced by different macronutrient compositions.

Nevertheless, the interplay between metabolic conditions, hormonal responses and food intake is not well understood and especially little is known about the interplay of macronutrient intake, endocrine factors and absolute energy intake. Most studies examine the effect of diets and food composition on endocrine parameters, food consumption and weight loss. Essential parts of the puzzle, such as hormones regulating the size of the meal and the specific composition of macronutrients, might therefore be missing [21]. Also, the regulation of macronutrient intake in different metabolic situations and long-term deficits or excesses is largely unknown. It has been shown that there are efficient compensatory mechanisms that are unlikely to be explained by fluctuation of hormones with fast kinetics [22]. Adipokines with slower kinetic characteristics may play a major role in this context [23].

To address the questions of how energy deficits control our eating behaviour even if energy deficits have been partially removed by glucose ingestion was the aim of this study. Additionally, it was attempted to find out if adiponectin, one of the emerging major players in the understanding of the metabolic syndrome, plays a role in the regulation of non-acute food intake and its composition under the metabolic conditions that apply in our experiment.

To fill in some of the research gaps, we measured the amount and composition of food consumed after a period of fasting or non-fasting (control); and after acute metabolic energy deficits were at least partly compensated for by oral ingestion of a large load of carbohydrates. It was hypothesised that fasting might still have implications on total food intake after the partial compensation of energy shortage by ingestion of glucose and the resulting alterations of hormones concerned with glucose metabolism towards an anabolic status. To induce insulin and C-peptide levels as high as possible after the glucose administration in the fasting condition to evaluate their role on appetite control as clearly as possible, studies that showed fasting induced glucose intolerance were used to determine the length of the fasting period [24,25]. These studies usually used fasting periods around 70 hours. After consultation with the ethics committee of the University of Luebeck we decided that a fasting period of 42 hours would be ideal in terms of combining the aims of likelihood of producing high insulin and C-peptide levels, safety and practicability. Furthermore, it was hypothesised that an association of the proportions of consumed food with adiponectin levels might exist in this context. This study focussed on a limited array of hormones because they were predominantly important to assess the impact of the oral glucose administration, due to reasons of practicability and the available means.

## Materials and methods

### Participants

24 healthy male participants of normal weight and without metabolic disease were enrolled for the study [age (mean ± SEM): 24.5 ± 0.6 years; body mass index (mean ± SEM): 23.4 ± 0.3 kg/m^2^]. Participants were not on any medication and had a regular self-reported sleep-wake cycle for 6 weeks before participating in the experiment. Individuals with acute or chronic diseases, drug abuse including alcohol (> 5 drinks per week) and smoking or exceptional physical activity were excluded. We also excluded individuals with special eating behaviours, e.g. vegetarians and vegans. The night before the experiment, the participants were asked to go to bed at approximately 11 p.m. and to avoid any exhausting physical activity. The study abided by the Declaration of Helsinki and was approved by the Ethics Committee of the University of Lübeck. All subjects gave written informed consent.

### Experimental setting

A within subject design with two conditions was used. Half of the participants started with the condition of total caloric deprivation (fasting condition = FAST) and the other half with the control condition (non-fasting = EAT). Conditions were spaced exactly 7 days apart.

Experiments started on the first day at 8 a.m. with participants arriving at the sleep laboratory of the Department of Psychiatry which is equipped to host long-term studies. For the fasting condition, participants were instructed to refrain from eating from 11 p.m. the evening before. In the control condition participants were provided with a standardised meal by the study team. Subsequently, an antecubital venous catheter was inserted for blood samples and half an hour later the first blood samples were obtained.

Afterwards, participants in the non-fasting condition had a standardized breakfast (2240 kcal, 14% proteins, 46% fat, 40% carbohydrates) followed by regular standardized meals (on average: 1320 kcal, 17% proteins, 31% fat, 51% carbohydrates); and during these meals, further blood samples were obtained. Overall 20 blood samples were obtained per condition over the two days of the experiment (blood samples were taken at 08:45 a.m., 10:00 a.m., 12:45 p.m., 02:00 p.m., 04:00 p.m., 06:00 p.m., 06:45 p.m., 08:00 p.m. and 10:00 p.m. on the first day and 08:45 a.m., 10:00 a.m., 11:45 a.m., 01:00 p.m., 01:35 p.m., 02:15 p.m., 02:45 p.m., 03:15 p.m., 03:45 p.m., 04:15 p.m. and 04:45 p.m. on the second day in both conditions). Participants were provided with oral glucose (polysaccharides dissolved in 300 mg of water that are broken down to 75g of glucose in the intestines; Accu-Chek Dextrose O.G.-T. 300 ml, Roche Diagnostics, ELISA, Indianapolis, IN, USA) at 1:30 p.m..

A standardized buffet was provided at 05:00 p.m. with a wide variety of offered foods and drinks. The participants were allowed to choose freely for one hour [26]. Food intake was measured and macronutrient composition as well as overall energy intake could be calculated for each individual. A list with detailed information about the food offered in the buffet can be found in the supplementary material S5 Dataset.

The experiment ended at 6 p.m. with the end of the buffet. The subjects in the fasting condition spent 42 hours in total without any caloric intake. No blood samples were obtained during the night-time to keep disturbance of physiological rhythms as minimal as possible. The subjects stayed at the study venue over the whole time of the experiment in single rooms and spend the night there as well with members of the study team being present over the whole time.

### Assays

Blood samples were centrifuged immediately after they were obtained, and the supernatant was stored at -80° C. The measurement of all hormones took place at the same point of time to avoid inter-assay variabilities. Blood serum and EDTA (Ethylenediaminetetraacetic acid) plasma were used to measure the hormone levels through an immunoassay. The ACTH-assay (Roche Diagnostics, ELCIA, Indianapolis, IN, USA) had a measuring range of 0.220–440 pmol/L, an intra-assay coefficient of variation (CV) of < 2.4% and an inter-assay CV of < 4.2%. The C-peptide-assay had a measuring range of 0.003–13.3 nmol/L, an intra-assay CV of <4.6% and an inter-assay CV of < 5.0. The Insulin-assay had a measuring range of 1.39–6945 pmol/L, an intra-assay CV of < 2.8% and an inter-assay CV of < 4,9%. Cortisol had a measuring range of 0.5–1750 nmol/L, an intra-assay CV of < 2.9% and an inter-assay CV of < 4.7%. Adiponectin levels were measured with an Adiponectin ELISA (Immundiagnostik AG, Adiponectin total ELISA Kit, Bensheim, Germany) with an intra-assay CV of < 3.4% and inter-assay CV of < 6.3%.

### Statistical analysis

MATLAB 2015a and SPSS version 23 were used for data preparation and statistical analysis. All data is presented as mean values ± SEM. Comparison of consumed food composition was carried out with paired student t-tests. The area under the curve (AUC) of the time course of the blood concentration of adiponectin was calculated with trapezoidal assumptions for the whole time of the experiment. A multiple stepwise linear regression with the individual AUC-values of adiponectin as a dependent variable and the relative quantities of the consumed macronutrient fat, carbohydrates and protein in the buffet test as independent variables was performed for each condition. For the comparison of the hormonal levels and plasma glucose levels within the different conditions, factorial ANOVAs were performed for all timepoints (timepoint, tp 1–20) and split into two parts—before the administration of oral glucose (tp 1–13) and afterwards (tp 14–20) to control for differences in the phases of the experiment. Significance was assumed for p-values <0.05.

## Results

### Buffet

**Fig 1:**
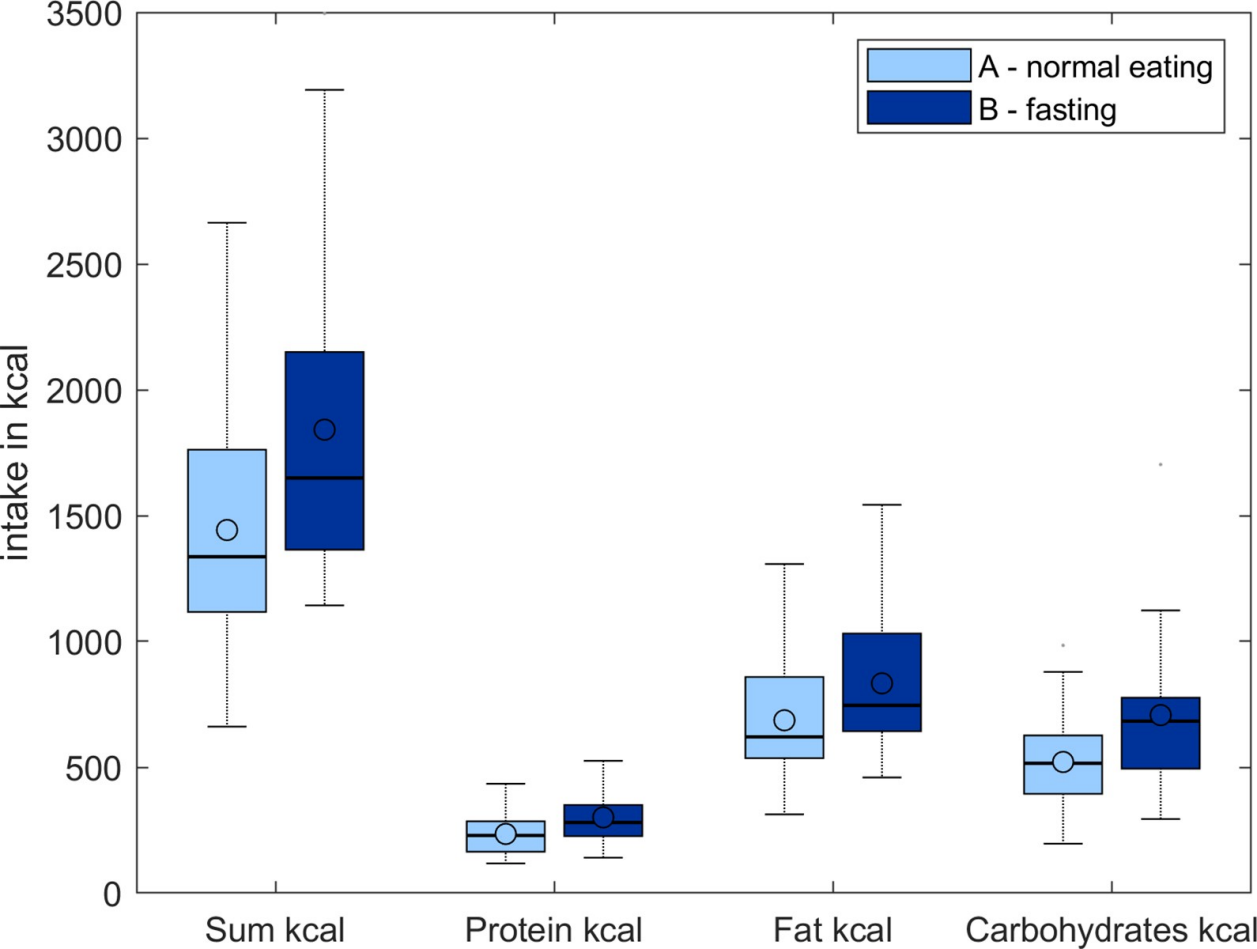
Food consumption during the buffet after 42 hours of fasting and the control condition. Box Plots of the total food consumption in the buffet scenario for both fasting and normal eating condition. The total amount of kilocalories and the overall amount of each macronutrient consumed differed significantly between the conditions.

The total number of kilocalories and the overall amount of each macronutrient consumed differed significantly between the conditions (Fig 1). Absolute food consumption was higher in the fasting group for all measured variables including the total amount of food (EAT: 1442.7±496.7 kcal; FAST: 1841.5±618.7 kcal; t(23) = 5.2, p<0.001), protein (EAT: 235.1±85.2 kcal; FAST: 300.9±103.6 kcal; t(23) = 5.4, p<0.001), fat (EAT: 686.7±262.5 kcal; FAST: 833.5±270.6 kcal; t(23) = 3.8, p = 0.001) and carbohydrates(EAT: 521±191.5 kcal; FAST: 707.2±302.5 kcal; t(23) = 3.7, p<0.001). However, the proportion of macronutrients in kilocalories showed no significant statistical difference between both conditions. Both conditions consumed the same percentage of proteins (16%, t(23) = 0.06, n.s.) and relatively similar percentages of fat (48% in the non-fasting condition and 45% in the fasting condition, t(23) = 1.3, n.s.) and carbohydrates (36% in the non-fasting condition and 38% in the fasting condition, t(23) = 1.2, n.s.).

### Hormones and blood glucose

**Fig 2:**
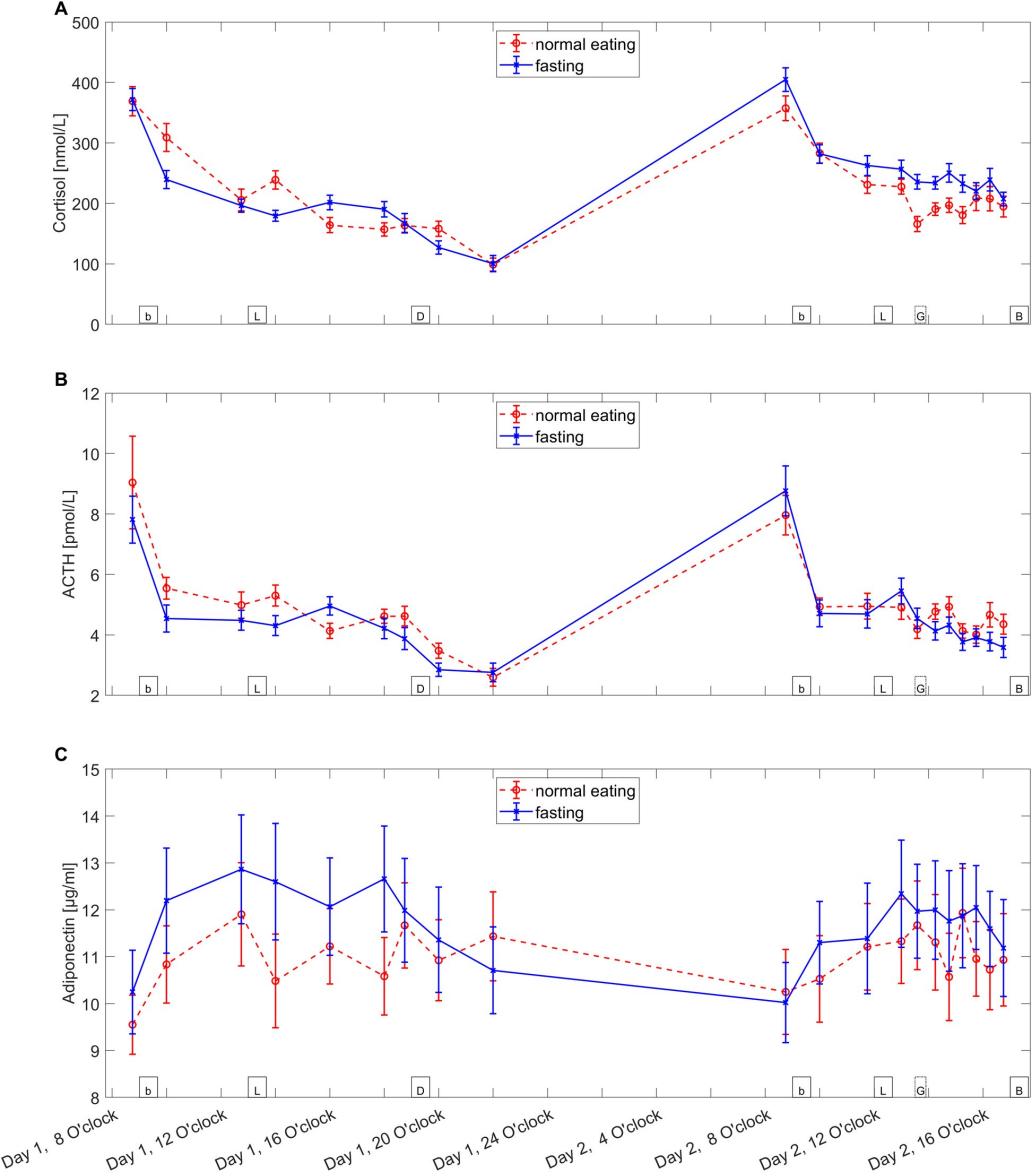
**Levels of cortisol (A), ACTH (B) and adiponectin (C).** The Figure shows the mean ± standard error of the mean for both fasting (blue line, crosses) and normal eating (red spotted line, circles) condition of the serum parameters during the experiment. Boxes on the bottom of the graph indicate the timepoints of meals (b = breakfast, L = lunch, D = Dinner, G = oral glucose administration, B = Buffet).

**Fig 3:**
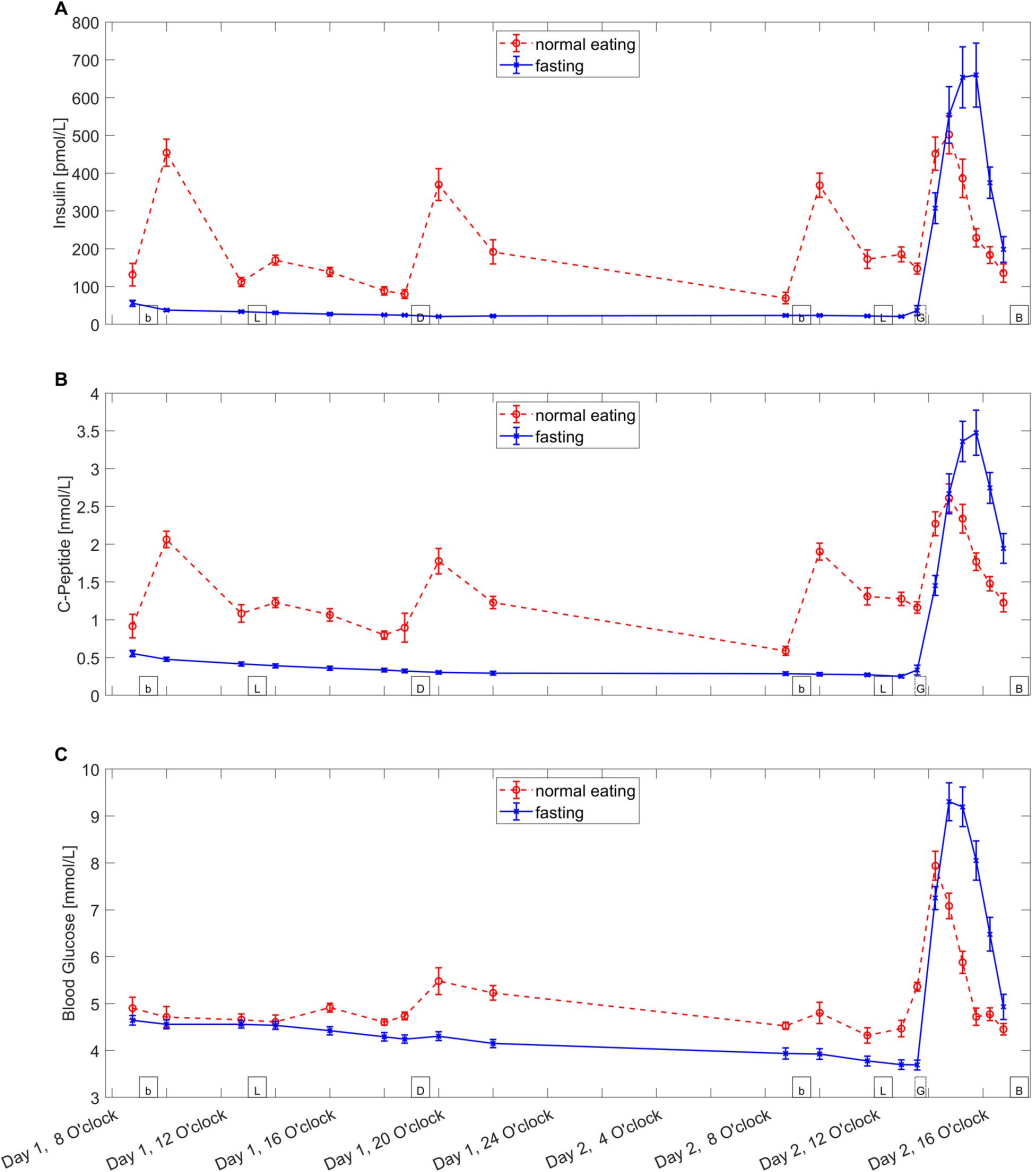
**Levels of insulin (A), C-peptide (B), blood glucose (C).** The Figure shows the mean ± standard error of the mean for both fasting (blue line, crosses) and normal eating (red spotted line, circles) condition of the serum parameters during the experiment. Boxes on the bottom of the graph indicate the timepoints of meals (b = breakfast, L = lunch, D = Dinner, G = oral glucose administration, B = Buffet).

Repeated measures ANOVAs including all 20 measurement timepoints revealed a main effect of conditions for insulin (F(1,23) = 27.7, p<0.001), C-peptide (F(1,23) = 62.2, p<0.001) and ACTH (F(1,23) = 4.7, p = 0.042) but not for cortisol (F(1,23) = 2.8, n.s.), glucose (F(1,23) = 1.2, n.s.) and adiponectin (F(1,23) = 3.3, n.s.). When the timepoints were separated into pre-oral glucose (tp 1–13) and post-oral glucose (tp 14–20), a significant difference was found for insulin (pre: F(1,23) = 371.9, p<0.001; post: F(1,23) = 12.6, p = 0.002), C-peptide (pre: F(1,23) = 331.8, p<0.001; post: F(1,23) = 19.2, p<0.001) and glucose (pre: F(1,23) = 30.5, p<0.001; post: F(1,23) = 42.8, p<0.001) in both sectors. Cortisol only showed significant differences in the post-oral glucose sector (pre: F(1,23) = 0.024, n.s.; post: F(1,23) = 7.6, p = 0.01). Both adiponectin (pre: F(1,23) = 2.8, p = 0.11; post: F(1,23) = 2.9 p = 0.1) and ACTH (pre: F(1,23) = 2.4 p = 0.14; post: F(1,23) = 2.9, p = 0.1) did not show any significant differences in either sector. Figs 2 and 3 show the time courses of all measured serum parameters.

### Correlations of adiponectin and food intake

A significant regression equation was found for the predictor relative protein content in the control condition (F(1,22) = 6.4, p = 0.19) with an R^2^ of 0.22 when the AUC-values of adiponectin were used as a dependent variable. No significant regression equation was found for the fasting condition.

## Discussion

This study compared food intake after either fasting or non-fasting periods when an acute caloric deficit was partially removed by oral glucose towards the end of the experiment. Whereas the total quantity of food ingested differed between conditions, the relative proportion of each macronutrient consumed did not. The relative amount of protein intake in the non-fasting condition correlated positively with the AUC-value of adiponectin throughout the experiment.

The observed increase in energy intake following a period of energy restriction has been described before [22,27,28] and appears to be intuitively the expected result. These studies suggest that there is a compensatory increase in overall energy intake after a period of caloric restriction as opposed to when individuals are not fasted. No previous study determined whether similar results would be obtained if the acute caloric deficit was compensated by the means of administering a large quantity of carbohydrates. Also the question whether the relative proportion of macronutrients consumed changes between fasting and non-fasting conditions has not been tackled previously.

Studies in obese subjects suggested that a compensatory increase in energy intake after a short energy restricted period does not occur [27,29], whereas data obtained in normal weight individuals tend to find an overall compensatory increase in energy intake after energy restriction thus corroborating the current results. This current experiment extended previous results by showing that food ingestion was increased after fasting in spite of application of oral glucose. The overall energy deficit of the body appeared to be driving overall energy consumption, even though insulin, C-peptide and serum glucose had higher levels in the fasting conditions at this time than in the control-condition. These higher levels reflect the well described phenomenon of “post-fasting glucose intolerance” [25,24]. In our case, this phenomenon shows that these parameters are unlikely to be the only factors providing the brain with feedback about the energy status of the body.

The relative macronutrient proportions of the ad libitum consumed food were not significantly different in between the conditions. We stress the fact that the results of the assessment of the macronutrient composition were only assessed in the context of the pre-meal glucose drink. The administration of the glucose was necessary to test for the main outcome of overall energy intake after a 42 caloric restriction after partial compensation with carbohydrates. Since there was no additional non-replenishment control group the influence of the glucose on the composition of food can’t be determined by this experiment. Further research is needed to confirm this finding and to determine whether the administered glucose might have affected this measurement as it has been shown that administering carbohydrates before a meal can have an influence on food choice. It has been shown before that carbohydrates can increase subsequent food consumption [30]. Thus, further evaluation of the influence a carbohydrate enriched diet has on food choice is needed. Furthermore, there are other possibilities why the food choice was driven into a certain direction. Some of the food items might have been more appetible than others due to their size or looks. Also, parts of the food were offered in a heated state, which might have directed attention towards these food items. Nevertheless, the stability of the proportions of food consumed under the different metabolic conditions remains noteworthy.

Adiponectin concentration did not differ between the conditions in this study. Due to the short but total caloric restriction, this result is in line with previous work showing that short total caloric restriction for 72 hours does not alter adiponectin levels over time [31]. It has also been shown that low caloric diets do not interfere with adiponectin levels for short [32] and intermediate [33] time periods providing that weight loss is minimal. Usually only long term fasting that results in meaningful weight loss results in rising adiponectin levels [34,35]; and recent work in rodents shows that caloric restriction for four weeks increases adiponectin expression on a cellular level [36]. Of note, declining adiponectin levels during fasting interventions have also been reported [37]. The duration of our study was too short to show alterations in levels of a hormone with slow kinetics such as adiponectin. It therefore supports current literature suggesting a very low diurnal and post-prandial biovariability of adiponectin [38], even when sweetened beverages were offered with normal meals [39].

The proportion of consumed proteins correlated with adiponectin levels only in the non-fasting condition. While existing evidence usually deals with the influence of dietary interventions on adiponectin levels [17,40,41], our finding may hint at a role of adiponectin level in food choice, i.e. macronutrient composition. Indeed, there is evidence adiponectin may take part in appetite regulation in the central nervous system [42,43]. However, whether adiponectin has an appetite stimulating [40] or attenuating role [41] remains controversial. The fact that our experiment did not show a difference in adiponectin levels during the experiment makes it less likely that the detected statistical association is derived from a physiological causal mechanism and overall harder to interpret the finding. No change in both adiponectin levels and macronutrient intake in between conditions while the associations only exists in one of the conditions does not leave any room for causal conclusions. However, different metabolic conditions might also alter the influence of endocrine signals on ingestive behaviour and adiponectin might exercise control differently over central nervous function in a fasting metabolic state than in a normally nourished one. Further research will be needed to evaluate the role of adiponectin in this context. Additionally, with regard to the relationship between adiponectin and protein intake, it has to be stressed that the direction of causality cannot fully determined by the current experiment. Although the adiponectin concentrations were measured before the buffet which might indicate that adiponectin was the cause and the protein intake the effect, as pointed out before, adiponectin has rather slow kinetic characteristics making reverse causality a possible option for the interpretation of the data.

Further limitations of this study include that neither women nor people outside an average weight range were included. Thus, these results might not extend to groups other than young, healthy normal weight men. Moreover, the oral glucose administration prior to the administration of the buffet test might have masked effects of fasting on food choice. Future studies might also explore to administer a more tailored quantity of glucose to compensate the energy deficit more precisely, which was not attempted in our study. This study measured only a limited number of hormones directly involved in glucose metabolism and one adipokine that has been shown before to be influenced by the composition of food. Further research into other endocrine signal parameters such as CKK, glucagon, GLP-1, ghrelin and leptin in this context will be needed to gain a more comprehensive understanding of quantity and quality of food intake.

## Supporting information

S1 Dataset Buffet fasting condition. Macronutrients consumed during the buffet after the fasting condition in kilocalories.
(TXT)

S2 Dataset Buffet control condition. Macronutrients consumed during the buffet after the control condition in kilocalories.
(TXT)

S3 Dataset Hormones fasting condition. Hormones and blood glucose measured at 20 timepoints each in the fasting condition.
(TXT)

S4 Dataset Hormones control condition. Hormones and blood glucose measured at 20 timepoints each in the control condition.
(TXT)

S5 Dataset Contents of buffet. Detailed list of contents of the standardised buffet the subjects could choose from at the end of the experiment.
(XLSX)

## References

[1] Jauch-Chara K, Oltmanns KM. Obesity–A neuropsychological disease? Systematic review and neuropsychological model. Prog Neurobiol [Internet]. 2014;114:84–101 Contents. Available from: 10.1016/j.pneurobio.2013.12.001 24394671

[2] Macdonald IA. A review of recent evidence relating to sugars, insulin resistance and diabetes. Eur J Nutr. 2016;55(s2):1–7. 27882410 10.1007/s00394-016-1340-8 PMC5174139

[3] Khan TA, Sievenpiper JL. Controversies about sugars: results from systematic reviews and meta-analyses on obesity, cardiometabolic disease and diabetes. Eur J Nutr. 2016;55(s2):1–19. 27900447 10.1007/s00394-016-1345-3 PMC5174149

[4] Blundell JE, Cooling J. Routes to obesity: phenotypes, food choices and activity. Br J Nutr. 2000;83 Suppl 1(May):S33–8. 10889790 10.1017/s0007114500000933

[5] Romieu I, Dossus L, Barquera S, Blottière HM, Franks PW, Gunter M, et al Energy balance and obesity: what are the main drivers? Cancer Causes Control. 2017;28(3):247–58. 10.1007/s10552-017-0869-z 28210884 PMC5325830

[6] González-Muniesa P, Mártinez-González M-A, Hu FB, Després J-P, Matsuzawa Y, Loos RJF, et al Obesity. Nat Rev. 2017;3. 10.1038/nrdp.2017.34 28617414

[7] Morton GJ, Meek TH, Schwartz MW. Neurobiology of food intake in health and disease. Nat Rev Neurosci [Internet]. 2014;15(6):367–78. Available from: 10.1038/nrn3745 24840801 PMC4076116

[8] Biebermann H, Castañeda TR, van Landeghem F, von Deimling A, Escher F, Brabant G, et al A role for β-melanocyte-stimulating hormone in human body-weight regulation. Cell Metab. 2006;3(2):141–6. 10.1016/j.cmet.2006.01.007 16459315

[9] Van Der Klaauw AA, Farooqi IS. The hunger genes: Pathways to obesity. Cell [Internet]. 2015;161(1):119–32. Available from: 10.1016/j.cell.2015.03.008 25815990

[10] Austin J, Marks D. Hormonal Regulators of Appetite. 2009;2009. 10.1016/j.appet.2008.09.015 PMC2777281 19946401

[11] Bernardini J, Kamara K, Castonguay TW. Macronutrient choice following food deprivation: Effect of dietary fat dilution. Brain Res Bull. 1993;32(5):543–8. 8221150 10.1016/0361-9230(93)90305-u

[12] Mcvay MA, Jeffreys AS, King HA, Olsen MK, Voils CI, Yancy WS. The relationship between pretreatment dietary composition and weight loss during a randomised trial of different diet approaches. J Hum Nutr Diet. 2015;28(s2):16–23. 10.1111/jhn.12188 24251378

[13] Abete I, Astrup A, Martínez JA, Thorsdottir I, Zulet MA. Obesity and the metabolic syndrome: Role of different dietary macronutrient distribution patterns and specific nutritional components on weight loss and maintenance. Nutr Rev. 2010;68(4):214–31. 10.1111/j.1753-4887.2010.00280.x 20416018

[14] Hall KD, Guo J. Obesity Energetics: Body Weight Regulation and the Effects of Diet Composition. Gastroenterology [Internet]. 2017;152(7):1718–1727.e3. Available from: http://www.ncbi.nlm.nih.gov/pubmed/28193517%0Ahttp://linkinghub.elsevier.com/retrieve/pii/S001650851730152X 10.1053/j.gastro.2017.01.052 28193517 PMC5568065

[15] Agus MSD, Swain JF, Larson CL, Eckert EA, Ludwig DS. Dietary composition and physiologic adaptations to energy restriction. Am J Clin Nutr. 2000;71(4):901–7. 10.1093/ajcn/71.4.901 10731495 PMC2905862

[16] Rajaie S, Azadbakht L, Saneei P, Khazaei M, Esmaillzadeh A. Comparative effects of carbohydrate versus fat restriction on serum levels of adipocytokines, markers of inflammation, and endothelial function among women with the metabolic syndrome: A randomized cross-over clinical trial. Ann Nutr Metab. 2013;63(1–2):159–67. 10.1159/000354868 24021709

[17] Summer SS, Brehm BJ, Benoit SC, D’Alessio D a. Adiponectin Changes in Relation to the Macronutrient Composition of a Weight-Loss Diet. Obesity. 2011;19(11):2198–204. 10.1038/oby.2011.60 21455123

[18] Dougkas A, Östman E. Protein-Enriched Liquid Preloads Varying in Macronutrient Content Modulate Appetite and Appetite-Regulating Hormones in Healthy Adults. J Nutr [Internet]. 2016;146(3):637–45. Available from: http://jn.nutrition.org/content/146/3/637.abstract 10.3945/jn.115.217224 26791555

[19] Ebbeling CB, Swain JF, Feldman H a, Wong WW, Hachey DL, Garcia-lago E, et al Effects of Dietary Composition During Weight Loss Maintenance: A Controlled Feeding Study. J Am Med Assoc. 2012;307(24):2627–34. 10.1001/jama.2012.6607 PMC3564212 22735432

[20] Stimson RH, Johnstone AM, Homer NZM, Wake DJ, Morton NM, Andrew R, et al Dietary macronutrient content alters cortisol metabolism independently of body weight changes in obese men. J Clin Endocrinol Metab. 2007;92(11):4480–4. 10.1210/jc.2007-0692 17785367

[21] Dulloo AG, Jacquet J, Montani J-P. How dieting makes some fatter: from a perspective of human body composition autoregulation. Proc Nutr Soc. 2012;71(3):379–89. 10.1017/S0029665112000225 22475574

[22] Anderson ML, Matsa DA. Are restaurants really supersizing America? Am Econ J Appl Econ. 2011;3(1):152–88.

[23] Farooqi IS, Bullmore E, Keogh J, Gillard J, O’Rahilly S, Fletcher PC. Leptin regulates striatal regions and human eating behavior. Science. 2007;317(5843):1355 10.1126/science.1144599 17690262 PMC3838941

[24] Johnson N a, Stannard SR, Rowlands DS, Chapman PG, Thompson CH, O’Connor H, et al Effect of short-term starvation versus high-fat diet on intramyocellular triglyceride accumulation and insulin resistance in physically fit men. Exp Physiol [Internet]. 2006;91(4):693–703. Available from: http://www.ncbi.nlm.nih.gov/pubmed/16627573 10.1113/expphysiol.2006.033399 16627573

[25] Frank P, Katz A, Andersson E, Sahlin K. Acute exercise reverses starvation-mediated insulin resistance in humans. AJP Endocrinol Metab [Internet]. 2013;304(4):E436–43. Available from: http://ajpendo.physiology.org/cgi/doi/10.1152/ajpendo.00416.2012 10.1152/ajpendo.00416.2012 23269410

[26] Hallschmid M, Jauch-Chara K, Korn O, Molle M, Rasch B, Born J, et al Euglycemic Infusion of Insulin Detemir Compared With Human Insulin Appears to Increase Direct Current Brain Potential Response and Reduces Food Intake While Inducing Similar Systemic Effects. Diabetes [Internet]. 2010;59(4):1101–7. Available from: http://www.ncbi.nlm.nih.gov/pubmed/20068139%0Ahttp://www.pubmedcentral.nih.gov/articlerender.fcgi?artid=PMC2844819%0Ahttp://diabetes.diabetesjournals.org/cgi/doi/10.2337/db09-1493 20068139 10.2337/db09-1493 PMC2844819

[27] Clayton DJ, Creese M, Skidmore N, Stensel DJ, James LJ. No effect of 24 h severe energy restriction on appetite regulation and ad libitum energy intake in overweight and obese males. Int J Obes [Internet]. 2016;40(11):1662–70. Available from: http://www.nature.com/doifinder/10.1038/ijo.2016.106 10.1038/ijo.2016.106 27339607

[28] Mars M, De Graaf C, De Groot LCPGM, Kok FJ. Decreases in fasting leptin and insulin concentrations after acute energy restriction and subsequent compensation in food intake. Am J Clin Nutr. 2005;81(3):570–7. 10.1093/ajcn/81.3.570 15755824

[29] Chowdhury EA, Richardson JD, Tsintzas K, Thompson D, Betts JA. Effect of extended morning fasting upon ad libitum lunch intake and associated metabolic and hormonal responses in obese adults. Int J Obes [Internet]. 2016;40(2):305–11. Available from: http://www.nature.com/doifinder/10.1038/ijo.2015.154 10.1038/ijo.2015.154 PMC4753359 26278005

[30] Bowen J, Noakes M, Trenerry C, Clifton PM. Energy intake, ghrelin, and cholecystokinin after different carbohydrate and protein preloads in overweight men. J Clin Endocrinol Metab. 2006;91(4):1477–83. 10.1210/jc.2005-1856 16434457

[31] Merl V, Peters A, Oltmanns KM, Kern W, Born J, Fehm HL, et al Serum adiponectin concentrations during a 72-hour fast in over- and normal-weight humans. Int J Obes (Lond) [Internet]. 2005;29(8):998–1001. Available from: 10.1038/sj.ijo.0802971%5Cnhttp://www.ncbi.nlm.nih.gov/pubmed/15917861 15917861

[32] Imbeault P, Pomerleau M, Harper ME, Doucet E. Unchanged fasting and postprandial adiponectin levels following a 4-day caloric restriction in young healthy men. Clin Endocrinol (Oxf) [Internet]. 2004;60(4):429–33. Available from: http://www.ncbi.nlm.nih.gov/pubmed/15049956 15049956 10.1111/j.1365-2265.2004.01997.x

[33] Anderlová K, Kremen J, Dolezalova R, Housova J J. The influence of very-low-calorie diet on serum leptin, soluble leptin receptor, adiponectin and resistin levels in obese women. Physiol Res. 2006;55(3):277–83. 16083306 10.33549/physiolres.930779

[34] Imbeault P. Environmental influences on adiponectin levels in humans. Appl Physiol Nutr Metab [Internet]. 2007 6 [cited 2017 Jun 5];32(3):505–11. Available from: http://www.nrcresearchpress.com/doi/abs/10.1139/H07-017 17510690 10.1139/H07-017

[35] Kotidis E V, Koliakos GG, Baltzopoulos VG, Ioannidis KN, Yovos JG, Papavramidis ST. Serum ghrelin, leptin and adiponectin levels before and after weight loss: comparison of three methods of treatment—a prospective study. Obes Surg. 2006;16(11):1425–32. 10.1381/096089206778870058 17132406

[36] Ding Q, Ash C, Mracek T, Merry B, Bing C. Caloric restriction increases adiponectin expression by adipose tissue and prevents the inhibitory effect of insulin on circulating adiponectin in rats. J Nutr Biochem [Internet]. 2012;23(8):867–74. Available from: 10.1016/j.jnutbio.2011.04.011 21852089

[37] Wolfe BE, Jimerson DC, Orlova C, Mantzoros CS. Effect of dieting on plasma leptin, soluble leptin receptor, adiponectin and resistin levels in healthy volunteers. Clin Endocrinol (Oxf). 2004;61(3):332–8. 15355449 10.1111/j.1365-2265.2004.02101.x

[38] Shand B, Elder P, Scott R, Frampton C, Willis J. Biovariability of plasma adiponectin. Clin Chem Lab Med [Internet]. 2006 1 1 [cited 2017 Jun 5];44(10):1264–8. Available from: https://www.degruyter.com/view/j/cclm.2006.44.issue-10/cclm.2006.227/cclm.2006.227.xml 10.1515/CCLM.2006.227 17032140

[39] Swarbrick MM, Havel PJ. Physiological, pharmacological, and nutritional regulation of circulating adiponectin concentrations in humans. Metab Syndr Relat Disord. 2008;6(2):87–102. 10.1089/met.2007.0029 18510434 PMC3190268

[40] Kubota N, Yano W, Kubota T, Yamauchi T, Itoh S, Kumagai H, et al Adiponectin Stimulates AMP-Activated Protein Kinase in the Hypothalamus and Increases Food Intake. Cell Metab. 2007;6(1):55–68. 10.1016/j.cmet.2007.06.003 17618856

[41] Shklyaev S, Aslanidi G, Tennant M, Prima V, Kohlbrenner E, Kroutov V, et al Sustained peripheral expression of transgene adiponectin offsets the development of diet-induced obesity in rats. Proc Natl Acad Sci U S A. 2003;100(24):14217–22. 10.1073/pnas.2333912100 14617771 PMC283572

[42] Kadowaki T, Yamauchi T, Kubota N. The physiological and pathophysiological role of adiponectin and adiponectin receptors in the peripheral tissues and CNS. FEBS Lett. 2008;582(1):74–80. 10.1016/j.febslet.2007.11.070 18054335

[43] Dridi S, Taouis M. Adiponectin and energy homeostasis: consensus and controversy. J Nutr Biochem [Internet]. 2009;20(11):831–9. Available from: 10.1016/j.jnutbio.2009.06.003 19716279

